# Superiority of a Treat-to-Target Strategy over Conventional Treatment with Fixed csDMARD and Corticosteroids: A Multi-Center Randomized Controlled Trial in RA Patients with an Inadequate Response to Conventional Synthetic DMARDs, and New Therapy with Certolizumab Pegol

**DOI:** 10.3390/jcm8030302

**Published:** 2019-03-03

**Authors:** Ruediger B. Mueller, Michael Spaeth, Cord von Restorff, Christoph Ackermann, Hendrik Schulze-Koops, Johannes von Kempis

**Affiliations:** 1 Division of Rheumatology and Immunology, Kantonsspital St. Gallen, 9007 St. Gallen, Switzerland; johannes.vonKempis@kssg.ch; 2 Division of Rheumatology and Clinical Immunology, Department of Internal Medicine IV, Ludwig-Maximilians-University Munich, 80336 Munich, Germany; Hendrik.Schulze-Koops@med.uni-muenchen.de; 3 Division of Rheumatology, Medical University Department, Kantonsspital Aarau, CH-5001 Aarau, Switzerland; 4 Division of Rheumatology, Spital Linth, 8730 Uznach, Switzerland; michael.spaeth@spital-linth.ch; 5 Medical practice, 8708 Männedorf, Switzerland; vonrestorff@hin.ch; 6 Medical practice, 9495 Triesen, Liechtenstein; ackermann.c@gmx.net

**Keywords:** rheumatoid arthritis, treat-to-target, certolizumab pegol, csDMARDs, glucocorticoids, intra-articular injections, DAS 28, ACR response, HAQ-DI

## Abstract

Background: Treatment of rheumatoid arthritis (RA) includes the use of conventional (cs), biologic (b) disease-modifying anti-rheumatic drugs (DMARDs) and oral, intramuscularly, intravenous, or intraarticular (IA) glucocorticoids (GCs). In this paper, we analysed whether a treat-to-target (T2T) strategy optimizing csDMARD, oral, and IA-GC treatment as an adjunct new therapy to a new certolizumab pegol (CZP) therapy improves the effectivity in RA patients. Methods: 43 patients with active RA (≥6 tender, ≥6 swollen joints, ESR ≥ 20 mm/h or CRP ≥ 7mg/L) despite csDMARD treatment for ≥ 3 months and naïve to bDMARDs were randomized to CZP (200 mg/2 weeks after loading with 400 mg at weeks 0–2–4) plus a treat-to-target strategy (T2T, *n* = 21), or to CZP added to the established csDMARD therapy (fixed regimen, *n* = 22). The T2T strategy consisted of changing the baseline csDMARD therapy (1) SC-methotrexate (dose: 15 ≥ 20 ≥ 25 mg/week, depending on the initial dose) ≥ leflunomide (20 mg/d) ≥ sulphasalazine (2 × 1000 mg/d) plus (2) oral GCs (prednisolone 20–15–12.5–10–7.5–5–2.5–0 mg/d tapered every five days) and (3) injections of ≤5 affected joints with triamcinolone. DMARD modification and an addition of oral GCs were initiated, depending on the achievement of low disease activity (DAS 28 < 3.2). The primary objective was defined as the ACR 50 response at week 24. Results: ACR 50 was achieved in 76.2% of the T2T, as compared to 36.4% of the fixed regimen patients (*p* = 0.020). ACR 20 and 70 responses were achieved in 90.5% and 71.4% of the T2T patients and 59.1% and 27.3% of the fixed regimen patients, respectively (*p* = 0.045 and *p* = 0.010, respectively). The adverse event rate was similar for both groups (T2T *n* = 51; fixed regimen *n* = 55). Conclusion: Treat-to-target management with the optimization of csDMARDs, oral, and IA-GCs of RA patients in parallel to a newly established CZP treatment was safe and efficacious in comparison to a fixed regimen of csDMARDs background therapy.

## 1. Introduction

Present treatment strategies for rheumatoid arthritis (RA) use conventional synthetic (cs), targeted synthetic (ts), and bDMARDs (biological disease modifying drugs). All of these classes of drugs have been shown to halt disease progression to a certain extent in clinical studies [1,2,3,4,5,6,7]. In general, therapy of RA is initiated with csDMARDs with or without concomitant glucocorticoids (GCs). If disease activity is not controlled under csDMARD treatment, ts or bDMARDs are added to treatment, as described, e.g., the EULAR (European League Against Rheumatism) guidelines [8]. In real life, a physician has more options than just adding the new ts/bDMARD. They can modify or optimize the therapy with concomitant csDMARDs, and oral or IA-GC (intraarticular glucocorticoids) can be added to the treatment regimen. Thus, a new therapeutic agent can be embedded in a whole strategy with parallel optimization of the csDMARD and GC treatment. In clinical studies with new compounds, on the other hand, the drug under examination is, in general, tested with a more or less fixed regimen of the pre-study csDMARDs, mostly methotrexate (MTX), and of GCs.

Strategic trials for the treatment of RA are rare [9,10,11,12]. In the TICORA (Tight Control of Rheumatoid Arthritis) trial [13], patients with early RA were treated, in the tight control arm, with a therapeutic strategy consisting of monthly visits, optimization of the therapeutic strategy (csDMARDs), and joint injections with GCs (triamcinolone acetonide). Not only was the efficacy, as measured by ACR (American College of Rheumatology) 20/50/70 responses, significantly higher in the patients in the tight control arm of the trial, as compared to the conventionally treated patients in the control arm of the study, but the rate of adverse events was also lower. A similar effect was repeated in the Camera study, where intensive treatment determined by a computerized decision program was superior to a conventional strategy [10]. This program forced the treating physician to increase the MTX dose and, as further escalation, add ciclosporine in early RA patients on a monthly basis, depending on the therapeutic response. This approach was shown to be superior to a conventional strategy with visits every three months. Goekoop-Ruiterman et al. demonstrated in the BeSt study that a treat-to-target approach was only superior with a step up to a bDMARD as compared to switching or adding more csDMARDs. Similarly, as shown in the Guepard study, the therapeutic efficacy under the guidance of tight control with MTX and step up to adalimumab was equally effective as compared to initial combination therapy of MTX and adalimumab after 12 months [14]. An ultrasound-guided step up of a therapeutic algorithm in early arthritis patients of the TaSer and the ARCTIC (Aiming for Remission in rheumatoid arthritis: a randomised trial examining the benefit of ultrasound in a Clinical TIght Control regimen) study did not lead to a significant amelioration of the clinical findings as compared to therapeutic escalation based on clinical assessment [11,12]. All these studies follow the idea of therapeutic escalation from cs to bDMARDs starting early in the course of the disease. In the EULAR recommendations, a therapeutic adjustment including the “optimization of csDMARDs dose or route of administration [15] or intra-articular injections of GCs” is recommended [8]. The European guidelines’ authors state that they did not want to “capture several current ways of GC application” independently of the dosage or route of application. In clinical trials, however, the doses of csDMARD are not always optimized. In the Oral Standard Study, the average dosage of MTX, which could not be increased during the trials, was 12.7 mg per week [16], and 16.5 mg per week in the Armada trial [17], suggesting that the dosage of csDMARD before and after the start of b or tsDMARDs had not been optimized. We hypothesize that the treating physicians may think that if one or more csDMARDs have failed, although the drug was not optimized, no additional effect of csDMARDs in the therapeutic algorithm can be expected. As in clinical trials, the doses of MTX are low; we estimate that csDMARDs are, in general, not optimized before or during a new b or tsDMARD treatment in real life, independently of what has been recommended by national or international organizations. During these trials [16,17], modification of the concomitant therapy with csDMARD and GCs was not allowed, as in almost every comparable trial.

Certolizumab pegol (CZP) is a PEGylated Fab’ fragment of a humanized anti-TNF (tumor necrosis factor) antibody with high affinity for TNF. CZP is an effective treatment, approved for moderate to severe RA in many countries, when given in combination with MTX, and has been demonstrated to be effective and safe in the treatment of RA in several phase III trials [18,19,20,21]. In the Rapid 1 trial, the ACR 20/50/70 responses to CZP were 59%, 37%, and 21%, respectively, at week 24 (week 52 was comparable) in combination with fixed regimen MTX and GC [20]. In comparison, in the TICORA study, an ACR 20/50/70 response was found in 91%, 84%, and 71%, respectively, of the patients in the tight control arm at 18 months.

Trials to date, involving CZP and other biologic agents, have primarily been controlled trials with placebo comparisons. This trial design has resulted in a lack of data concerning the use of bDMARDs in a setting closer to real-life, where treatment strategies are more adaptive based on the patient’s signs and symptoms. This trial was aimed at comparing the use of CZP in patients with moderate to severe RA, when administered in conjunction with an intensive, adaptive treatment program, versus a fixed regimen approach, as seen in the previous clinical trials. We hypothesized that an adaptive treat-to-target (T2T) strategy, in parallel to a new initiation of CZP, will lead to an improved outcome of RA patients with active disease despite csDMARD treatment, as compared to patients treated with a regimen of fixed csDMARDs and GC.

## 2. Methods

### 2.1. Patients

This study was conducted at four sites in Switzerland and Liechtenstein. Between March 2014 and December 2017, we recruited patients above 18 years of age suffering from RA and fulfilling the 2010 ACR/EULAR criteria [22] (NCT02293590). All patients had active disease despite csDMARD treatment prior to the baseline for ≥three months, at a stable dose for ≥4 weeks for oral glucocorticoids ≤10 mg daily prednisolone or equivalent and csDMARDs, defined by ≥6 tender and ≥6 swollen joint defined active disease out of the 66/68 joint count and an ESR (erythrocyte sedimentation rate) ≥ 20 mm/h or a CRP (C reactive protein) ≥ 7 mg/L. Key exclusion criteria included: previous treatment with a bDMARD; missing anti-conception in fertile patients; untreated, active, or latent bacterial (e.g., tuberculosis) or viral infections; or malignant diseases within the last five years.

This study was conducted in compliance with the ethical principles described in the Declaration of Helsinki and Good Clinical Practice. Approval of the Institutional Review Boards designated by each study site was obtained. All patients signed a written informed consent form.

### 2.2. Procedures

All patients were started on CZP (400 mg subcutaneously at weeks 0-2-4, and then 200 mg every two weeks to week 24). Patients were randomly assigned to either the T2T group or the group with the fixed regimen of the background treatment with csDMARDs and GCs. Both groups were started on CZP after randomization. Randomization at week 0 was performed centrally using the Secutrial system (interActive SystemsGmbH, Berlin, Germany, also used as clinical research form). This system used for assigning eligible subjects to a treatment regimen was based on a predetermined schedule for minimization. Minimization was conducted with respect to DAS (disease activity score, based on 28 joints) 28 ≥ 5.1 vs. <5.1, age ≥ 55 vs. <55, and male vs. female gender.

Patients were reviewed for their disease activity at weeks 4, 8, 12, 18, and 24 to determine their treatment strategy and to document the proportion of patients classified as treatment responders. The efficacy assessments included the number of swollen and tender joints (66/68 joint count); DAS 28; the patient’s global assessment of disease activity and pain; the physician’s global assessment of disease activity; CRP, ESR, and HAQ-DI (health assessment questionaire disability index) scores; and ACR 20/50/70 responses.

Efficacy as a basis for adaption of the therapeutic strategy during the trial (outlined below) was defined by a DAS 28 reduction of ≥1.2 since the last visit or achievement of LDA (low disease activity, DAS 28 ≤ 3.2). Therapy was modified if these criteria were not fulfilled. In the case of treatment-emergent adverse events, the DMARD could be tapered stepwise.

### 2.3. Therapeutic Strategies

#### 2.3.1. T2T csDMARD Strategy

At the study start and during the study, patients who did not reach a sufficient treatment response, as outlined in the previous chapter, or who developed treatment-emergent adverse events, were taken to the next step or drug according to the therapeutic algorithm. For example:

Step 1: 15 mg MTX/week (MTX was always employed SC (subcutaneous) in the T2T arm);

Step 2: 20 mg MTX/week;

Step 3: 25 mg MTX/week;

Step 4: 20 mg leflunomide/d;

Step 5: sulphasalazine (target dose 2 × 1000 mg/d).

**Figure 1:**
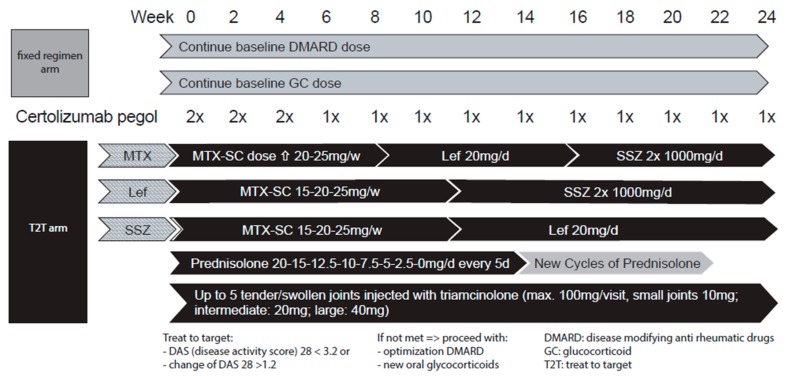
The treatment strategy arms: Flow diagram for treatment for escalation of disease-modifying anti-rheumatic therapy in RA patients. Patients were randomized into two arms: Fixed regimen (grey) or T2T (treat-to-target, black). Patients with both therapeutic arms were treated with certolizumab pegol (CZP). Fixed regimen arm: Baseline GCs and csDMARDs were continued at a stable dose throughout the study. T2T arm: Depending on the csDMARD used before the study (shown as a striped arrow), the csDMARD therapy was modified. In parallel, a cycle of oral GCs was initiated, and up to five tender and/or swollen joints were injected with triamcinolone. The response criteria for the patients in the T2T arm are listed below. If these response criteria were not met, therapy was modified according to the mechanism described, and a new cycle of oral GCs was initiated. Joint injections were always conducted independently of achieving the pre-set goal of the T2T strategy if they were tender and/or swollen. Lef, leflunomide; MTX-SC, subcutaneous methotrexate; SSZ, sulphasalazine.

If a drug of this therapeutic algorithm had already been stopped before study entry because of intolerance, this drug was left out, and csDMARD treatment was directly continued with the next drug. This algorithm is illustrated in Figure 1.

#### 2.3.2. T2T GC Strategy

At the study start, all T2T patients were started on an oral dose of prednisolone 20 mg once daily, which was then tapered down every five days at the following increments: 20 mg, 15.0 mg, 12.5 mg, 10 mg, 7.5 mg, 5 mg, 2.5 mg, and 0 mg once daily. If the DAS 28 had not improved by ≥1.2 or LDA was not reached at the subsequent visit, prednisolone was started again at 20 mg once daily, followed by the same tapering schedule as noted above.

Starting at week 0, up to five synovitic joints had to be injected. The maximum cumulative triamcinolone dose was 100 mg/visit.

Doses of triamcinolone and lidocaine for joint injections were:

- Small joints: metacarpophalangeal (MCP), proximal interphalangeal (PIP), metatarsophalangeal joints (MTP): distal interphalangeal joints (DIP), sternoclavicular joint, acromioclavicular joint, tarsus, and distal interphalangeal joints of the feet (IP): 10 mg triamcinolone;
- Intermediate joints: carpus, elbow, ankle: 20 mg triamcinolone with 0.5 mL 1% lidocaine; big joints: knee, shoulder, hip: 40 mg triamcinolone with 4 mL 1% lidocaine.

If more than five synovitic joints were found to be painful and/or tender, the physician selected the joints mostly affecting daily life activities based on the patient’s judgment.

#### 2.3.3. Fixed Regimen Strategy

Patients continued to receive their stable weekly dose of csDMARD and GC (≤10 mg prednisolone or equivalent), as noted at study entry, for the duration of the study (24 weeks, Figure 1).

### 2.4. Primary Objective

The primary objective was to determine the number of patients exhibiting at least a 50% improvement of the disease activity, as determined by the ACR 50 response following 24 weeks of treatment of CZP, when given as part of an intensive adaptive treatment plan (T2T), in comparison to a therapy with fixed, continued, csDMARDs and GCs with unchangeable doses (fixed regimen).

### 2.5. Anti-Drug Antibodies

Sera from patients were collected at baseline and at four and 24 weeks of the study. Samples at weeks 4 (because a first exposure to the drug was needed to measure CZP levels) and 24 were used to measure CZP drug levels. Samples taken at baseline and at week 24 were analysed for anti-drug antibodies. Analyses were conducted at Sanquin Research (Department of Immunopathology, Amsterdam, The Netherlands), as previously described [23].

### 2.6. Data and Statistical Methods

#### 2.6.1. Power Calculation

To detect an increase in ACR 50 from 37% assumed in the fixed regimen group [20] to 84% in the T2T group [13], 21 patients were required in each group, if a chi-square test was used, to achieve 90% power, if the two-sided significance level was 0.05.

#### 2.6.2. Statistical Analyses

Demographic variables and baseline characteristics of the disease activity were summarized using descriptive statistics. For ACR 20/50/70; DAS 28; and HAQ-DI, the chi-square test was used. Missing values on the ACR responses were calculated as “not reached” (worst-case analysis). Missing values of the DAS 28 and HAQ-DI were not imputed for the calculations (per protocol).

The chi-square test compared the proportion of the two groups of patients in remission. Time to DAS 28, CDAI (clinical disease activity index), and Boolean defined remission was summarized using Kaplan-Meier curves and compared using the log-rank test. The cumulative corticosteroid dose was compared for the two patient groups using the Mann-Whitney *U*-test. Safety outcomes, including treatment-emergent adverse events, were summarized in a table by type and severity and reported for all patients receiving at least one dose of CZP. Anti-drug antibodies and CZP levels were measured at the last visit, using the Mann-Whitney *U*-test.

## 3. Results

### 3.1. Patient Demographics/Characteristics

**Figure 2:**
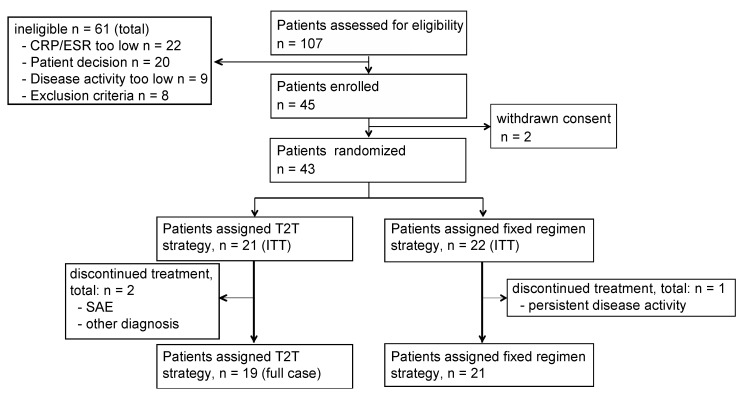
Patient disposition: T2T: treat-to-target, ITT: intention to treat, ESR: erythrocyte sedimentation rate, CRP: C reactive protein, SAE: serious adverse event.

We pre-screened 107 patients for the study (first patient in 27 March 2014; last patient out 22 December 2017); after exclusions and two withdrawn consents before randomization, 21 patients were assigned to the T2T, and 22 to the fixed regimen arm of the study. Of these, 19 patients (90.5%) in the T2T arm and 21 patients (95.5%) in the fixed regimen arm completed the 24-week study (Figure 2). Two patients dropped out of the T2T group: one because of an adverse event and one because the initial diagnosis of RA (inclusion criterion) had to be revised and a new primary diagnosis was established. One patient of the fixed regimen arm dropped out for persistent disease activity. Baseline characteristics and measures of disease activity in the two groups were balanced (Table 1). Mean DAS 28 (ESR) was 5.89 ± 0.98 for T2T and 6.16 ± 0.86 for fixed regimen patients. Small differences in baseline measures of disease activity were deemed not to be clinically relevant. Median RA duration (time from the first diagnosis) was approximately one year in both groups. Bone erosions on hands and/or feet were described in 26.3% of the T2T patients and 30.0% of the fixed regimen patients.

### 3.2. Clinical Responses

**Figure 3:**
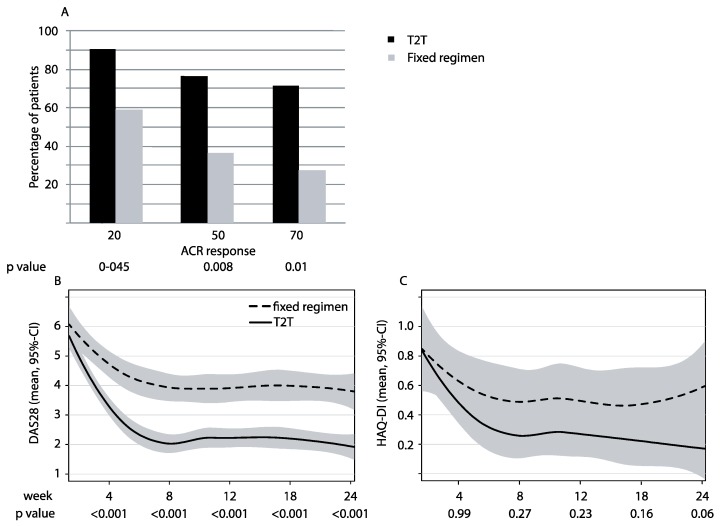
Response to treatment: (**A**) Percentage of patients achieving an ACR 20/50/70 response was demonstrated at week 24 (ITT analysis). T2T is shown in black, and fixed regimen in grey. (**B**) Average disease activity score in 28 joints (DAS 28) at baseline and during follow up until week 24. (**C**) Average health assessment questionnaire disability index (HAQ-DI) at baseline and during follow up until week 24. All data are shown for the two patient groups as means with the 95% confidence interval: T2T solid black line, and fixed regimen as a striped line; ACR: American college of rheumatology, ITT: intention to treat, T2T: treat to target.

Treatment of RA with CZP plus T2T background therapy significantly reduced signs and symptoms as compared with CZP plus fixed regimen background therapy. At 24 weeks, the ACR 50 response rate (primary endpoint) was achieved in 16 out of 21 T2T patients (76.2%), as compared to eight out of 22 fixed regimen patients (36.4%; Chi^2^: 5.355, *p* = 0.020, Figure 3A). ACR 20 and 70 responses were achieved in 90.5% and 71.4% of the T2T patients, and in 59.1% and 27.3% of the fixed regimen patients, respectively (*p* = 0.045 and *p* = 0.010, respectively, Figure 3A).

The improvement in disease activity, as assessed by DAS 28, developed more rapidly within the first four weeks of treatment and remained after that at sustained lower levels until week 24 (Figure 3B) in the CZP plus T2T treated patients, as compared to the patients treated with the fixed regimen co-therapy. In more detail, DAS 28 levels decreased to 2.62 and 1.91 at weeks 4 and 24 in the T2T group, as compared to 4.21 and 3.77 in the fixed regimen group, respectively (*p* < 0.001 for all time points, Figure 3B).

In parallel, higher remission rates were found for CZP and T2T treated patients independently of the definition of remission employed. DAS-defined remission (DAS 28 < 2.6) was found in 68.4% vs. 28.6% (Log-rank test *p* < 0.001), CDAI defined remission (CDAI ≤ 2.8) in 78.9% vs. 23.8% (Log-rank test *p* < 0.001), and Boolean remission in 47.4% vs. 19.1% (Log-rank test *p* = 0.031) of the T2T and fixed regimen therapy patients, respectively, at week 24 (data shown as Supplementary Materials).

### 3.3. Patient-Related Outcomes

The improvement in the HAQ-DI as a patient-related outcome developed more rapidly within the first four weeks of treatment, and remained after that at sustained lower levels until week 24 (Figure 3C) in the CZP plus T2T treated patients, as compared to the patients treated with the fixed regimen co-therapy. The mean reduction in HAQ-DI was markedly higher in the T2T group than in the fixed regimen group; however, significance was not reached (week 24: T2T 0.17 vs. fixed regimen 0.6, *p* = 0.06). Similarly, the changes in the patient’s pain score and in patient’s global assessment of disease activity were not significantly different at week 24 (Table 2).

### 3.4. Adverse Events

Of the 43 patients in this study, 18 (76.2%) patients in the T2T and 16 (72.7%) in the treatment fixed regimen reported at least one adverse event (AEs). The most common AEs were infections. Most reported AEs were of mild or moderate severity (98.1% T2T and 98.0% fixed regimen). Only three patients reported serious AEs (SAE: *n* = 2 T2T; *n* = 1 fixed regimen). The SAEs were: insufficiency of the adrenal gland, cardiovascular infarction (both SAEs were T2T), and bursitis infra-patellaris of the right knee (fixed regimen). All SAEs were resolved (Table 3). The most frequent AEs were infections. Thirteen T2T patients and 10 fixed regimen patients had infections during the study. In detail, we found bursitis (*n* = 1), a nail infection (*n* = 1), lip blisters (*n* = 3), pyelonephritis (*n* = 1), an upper respiratory infection (*n* = 3), and a urinary tract infection (*n* = 1) in fixed regimen patients and cutaneous mucositis (*n* = 1), parondontitis (*n* = 1), a gastrointestinal infection (*n* = 1), Herpes Labialis, (*n* = 1) mycosis (*n* = 1), pneumonia (*n* = 1), a skin infection (*n* = 1), and an upper airway infection (*n* = 6) in T2T patients.

### 3.5. Cumulative Steroid Dose

For the T2T group, the cumulative oral corticosteroid dose was calculated by counting the number of GC cycles: One patient required three cycles of oral GC, four patients two cycles, and all the other T2T patients only one cycle. In total, an average of 471.2 mg prednisolone (SD (standard deviation) ± 207.1) was used in the T2T patients. Additionally, 10.2 (mean, SD ± 7.1) joints were injected with triamcinolone per T2T patient. Furthermore, 7.2 small, 1.7 intermediate, and 1.0 large joints were injected per patient. In total, a mean of 149.1 mg triamcinolone (SD ± 109.3) was injected into the joints of T2T patients. This adds up to a cumulative average GC dose (equivalence dose prednisolone:triamcinolone = 1:1.2) in T2T patients of 666.9 mg (SD ± 309.5) prednisolone/patient or equivalent [24] over the whole study period.

Among the fixed regimen patients, three patients were continuously treated with 10 mg prednisolone/d, two with 7.5 mg/d, seven with 5 mg/d, two with 2.5 mg/d, and eight without concomitant prednisolone. This adds up to a cumulative average GC dose in fixed regimen patients of 792.5 mg prednisolone/patient or equivalent (SD ± 594.5) over the whole study period. The cumulative GC doses did not differ significantly between both groups (Mann-Whitney test, *p* = 0.723, Figure S1).

### 3.6. Therapeutic Changes of the csDMARD Protocol

Only two patients required two modifications of their baseline T2T csDMARD regimen. All other patient required only one optimization of the csDMARD. Two patients required a step up from 10 mg to 15 mg MTX/week, nine from 15 mg to 20 mg MTX/week, six from 20 to 25 mg MTX/week, two from 25 mg MTX/week to 20 mg leflunomide/d, three from 20 mg leflunomide/d to 15 mg MTX/week, and one from 20 mg leflunomide/d to 2 × 1000 mg sulphasalazine/day.

### 3.7. ADA (Anti Drug Antibodies) Antibodies

Blood serum CZP levels were analysed at week 4 and after 24 weeks and for anti-CZP antibodies at baseline and after 24 weeks. Anti-CZP antibodies were not detectable at baseline, but were detected after 24 weeks of treatment in 8/19 patients of the T2T group (42.1%) and in 10/19 patients of the fixed-dose group (52.6%). This difference in frequencies was not significant (chi^2^ = 0.106, *p* = 0.745). There was, however, a trend of lower anti-CZP antibodies levels in the T2T group (T2T: 139.1 ± 138.8 vs. fixed regimen: 197.1 ± 170.0 AU (arbitrary units)/mL, Mann-Whitney test, *p* = 0.439, Figure S2).

CZP levels decreased from week 4 (46.5 ± 19.4 mg/mL) to week 24 (29.5 ± 12.6 mg/mL, Wilcoxon signed-rank test, *p* < 0.001), but did not differ between treatment groups (Mann-Whitney test, week 4: *p* = 0.857, week 24: *p* = 0.977).

## 4. Discussion

Treat-to-target management with the optimization of csDMARDs, oral, and of IA-GC (T2T) in parallel to new CZP treatment was safe and led to a substantial decrease of disease activity, as compared to new CZP with a fixed regimen of csDMARDs and GCs, in RA patients with an inadequate response to previous csDMARDs.

The idea of treating to target has been around since the late 90s for the treatment of RA patients. The study employing a modification of csDMARDs and IA-GCs had much influence on how we treat RA to target nowadays [13]. The arrival of TNF antagonists and the other biologics had an even more profound impact on the treatment of RA. As mentioned in the introduction, the underlying principle of these T2T strategies is that if a class of drugs (e.g., csDMARDs) has failed to reach the target, mostly due to low disease activity or even remission, a step up to the next class of therapeutics (bDMARDs) is immediately necessary. Thus, an assumption is made that the csDMARD that has failed cannot add any additional efficacy to the treatment of this patient. The novelty of our protocol is that the therapeutic strategy is optimized by four parallel actions: first bDMARD (TNF antagonist), csDMARD with dose adjustment, and additional oral and IA-GCs. Adaptation of concomitant medication is generally not allowed in clinical phase II/III studies for a new compound in RA. The superiority of a combination of the treat-to-target idea, as suggested by the TICORA study, with the addition of a biologic treatment at a level that has never been reached for any biologic agents, in csDMARD-IR patients, is the real novelty of this study.

Over the last two decades, various compounds have been tested for efficacy and safety: combination therapy of csDMARDs, TNF antagonists, other biologic agents, and targeted synthetic DMARDs. Efficacy, as assessed by ACR response rates, was always comparable: 60% for ACR 20, 40% for ACR 50, and 20% for ACR 70, in MTX-incomplete, responding patients [16,20,25,26,27].

Every method available to improve an RA patient’s health, i.e., csDMARD dose adjustment, in addition to oral and IA-GCs and bDMARD, should be applied to improve these response rates. It seems likely that the effects demonstrated here for a T2T strategy in combination with a parallel start of CZP can be generalized for all bDMARDs and tsDMARDs in csDMARD-IR patients. Whether a similar effectiveness could also be achieved after an inadequate response to b or tsDMARDs would have to be confirmed in other trials with a design similar to ours.

This publication has several limitations: Firstly, the differences found for efficacy (ACR response, DAS28, CDAI) could not be demonstrated at a significance level for HAQ-DI and other patient-related outcomes (PROs), but a strong tendency towards superiority of the T2T-regimen was found. Little is known about the response of HAQ-DI in a treat-to-target clinical trial setting. The only publication with a comparable design, where Hirano et al. demonstrated a reduction of HAQ-DI from 1.4 to 1.0 when following the Brazilian therapeutic recommendations, had no control group and the observation period was much longer [28]. As a consequence, we did not have any data to calculate the power for significant changes in HAQ-DI for the design of our study. Considering that 84% of the T2T patients achieved an HAQ-DI < 0.5, and 65% of the patients with a fixed regimen, a sample size of two times 81 patients would have been necessary to reach a power of 0.8 with an alpha of 0.05. It has to be considered, that in the face of the impressive results regarding efficacy, we would have treated an additional 60 patients according to the fixed regimen protocol with a significantly lower clinical efficacy. In summary, we think that this would have been ethically controversial. Psychological effects of RA are well-described. Such phenomena may influence PROs [29,30]. Strand et al. [31] have described that PROs improve with CZP treatment. Interestingly, this improvement had the lowest correlation with DAS 28 or ACR 20 response levels. It seems likely that regression towards the mean may be a reason why the PROs in this study did not reflect the outstanding results of the efficacy parameters [32].

Secondly, changes in radiographic progression were not analysed. The reasons for this were the sample size and that the study was only planned for 24 weeks. Concerning these two restrictions, we did not expect to find any differences in the radiographic progression during the study. The low rate of radiographic progression usually found in comparable patient populations was another reason not to consider it as an outcome parameter: an increase of 0.4 Sharp units over only 52 weeks, or 0.2 over 24 weeks, was found in patients treated with CZP and MTX in the 0.2 units for RAPID 2 and 0.4 for RAPID 1.

Thirdly, a double blind, randomized study is superior to an open study like ours. On the other hand, studies with the combined therapeutic treat-to-target approaches, especially involving IA-GCs, are difficult to blind. In the TICORA [13] and CAMERA [10] studies, which, like our study, also used joint injections, the two therapeutic arms were not blinded.

## 5. Conclusions

In conclusion, this study shows that a T2T approach in the treatment of RA patients with an inadequate response to csDMARDs, using the full armamentarium of the therapeutic possibilities, has a high potential to improve disease activity in parallel to the start of a bDMARD, in our case, CZP. We feel strongly that this approach is transferable to the start of other b or tsDMARDs. We are aware of the fact that designing clinical studies for the demonstration of effectivity/effectiveness of new drugs with dynamic background therapies is difficult. This, however, should not influence our daily approach to ameliorate the situation for our patients.

## Figures and Tables

**Table 1:** Demographics and disease activity at screening.

	T2T (*n* = 21)	Fixed Regimen (*n* = 22)
Age (a, mean)	56.3 ± 15.4	56.8 ± 14.8
Gender (% female)	66.7%	63.6%
BMI (Kg/m^2^, mean)	28.6 ± 4.4	28.7 ± 5.6
ACPA pos. (%)	47.6%	47.6%
RF pos. (%)	66.7%	81.0%
Erosive disease (%, defined by treating physician)	26.3%	30.0%
Disease duration (a, median, range)	0.99, 3 months–10 years	0.85, 3 months–18 years
Concomittant DMARD at baseline	MTX 10 mg/w, *n* = 2 MTX 15 mg/w, *n* = 9 MTX 20 mg/w, *n* = 4 MTX 25 mg/w, *n* = 3 Lef 20 mg/d, *n* = 3	MTX 10 mg/w: *n* = 2 MTX 15 mg/w *n* = 6 MTX 20 mg/w *n* = 3 MTX 25 mg *n* = 5 MTX + HCQ, *n* = 2 15 mg/w + 200 mg/d; 20 mg/w + 400 mg/d SSZ *n* = 2 Lef *n* = 2
Concomitant GC (mean dose *, number of patients)	4.7 mg/d; *n* = 8	6.3 mg/d; *n* = 10
Disease activity score (DAS 28)	5.89 ± 0.98	6.16 ± 0.86
Tender joint score (0–68)	20.7 ± 10.3	23.2 ± 13.7
Swollen joint score (0–66)	18.9 ± 7.6	18.6 ± 10.9
Pain score (0–100)	65.3 ± 20.8	60.2 ± 21.0
Patient global assessment	70.1 ± 16.0	64.2 ± 16.9
Physician global assessment (0–100)	71.8 ± 8.8	67.0 ± 19.0
C-reactive protein (mg/L)	13.0 ± 16.2	17.1 ± 18.8
Erythrocyte sedimentation rate (mm/h)	28.7 ± 19.9	35.1 ± 25.2
Health assessment questionnaire score * (0–3)	0.84 ± 0.62	0.85 ± 0.64

* assessed on patients treated with glucocorticoids; Lef: leflunomide; SSZ: sulphasalazine; MTX: methotrexate; HCQ: hydroxychloroqine, T2T: treat to target, BMI: body mass index, ACPA: anti-citrullinated protein antibodies, RF: rheumatoid factor, pos.: positive, DMARD: disease modifying anti rheumatic drugs, GC: glucocorticoids, DAS: disease activity score.

**Table 2:** Improvement of ACR core components at week 24.

	T2T	Fixed Regimen	*p*-Value
Patient’s pain score	−53.34	−50.51	n.s.
Patient’s global assessment	−56.21	−49.59	n.s.
Physician’s global assessment	−64.62	−31.74	<0.001
Tender joint count (68)	−19.67	−9.9	<0.001
Swollen joint count (66)	−17.89	−8.76	<0.001
ESR (mm/h)	−12.83	−17.86	<0.001
CRP (mg/L)	−3.03	−7.64	<0.001
HAQ-DI	−0.68	−0.25	n.s.
CDAI	−33.81	−21.9	<0.001

T2T: treat to target, n.s.: not significant, ESR: erythrocyte sedimentation rate, CRP: C reactive protein, HAQ-DI: health assessment questionaire disability index, CDAI: clinical disease activity index.

**Table 3:** Adverse events.

	Fixed Regimen	T2T
Total (*n*)	51	55
Patients with AEs (*n*)	16	18
Intensity (mild/moderate/severe, *n*)	49/1/1	50/4/1
SAE (*n*)	1 *	2 **
Related (Not, unlikely/possibly/ probably/definitely, *n*)	22/3/21/4/1	33/3/19/0/1
Cardiovascular (*n*)	2	7
Dermatological (*n*)	6	4
Gastrointestinal (*n*)	3	4
General (*n*)	1	4
Hepatology (*n*)	2	1
Infection	10	13
Injection site reaction to CZP (*n*)	4	3
Injury (*n*)	5	2
Joint injection reaction (*n*)	-	3
Musculoskeletal (*n*)	4	3
Neurological (*n*)	11	10
Ophthalmological (*n*)	1	1
Psychology (*n*)	2	1

*n*: number; (S) AE: (serious) adverse events; CZP: Certolizumab pegol; * bursitis infrapatellaris; ** insufficiency of adrenal gland, cardiovascular infarction; T2T: treat to target.

## Supplementary Materials

The following are available online at https://www.mdpi.com/2077-0383/8/3/302/s1, Figure S1: Cumulative GC (glucocorticoids) dose during the study period. The average cumulative GC dose was calculated separately for patients treated with T2T or with fixed regimen. The average cumulative GC dose was standardized for prednisolone or equivalent, Figure S2: (A) CZP (certolizumab pegol) levels and were measured at week 4 and week 24. (B) CZP anti-drug antibodies were measured at the baseline and week 24. Patients treated with CZP + T2T are shown in dark grey and CZP + fixed regimen in light grey. Data are shown as medians with the 25th and 75th percentile and the standard deviation as error bars. Outliers are implemented as dots; w: week, AU/mL: arbitrary units per milliliter, T2T: treat to target.
