# Supplementary material for: Superiority of a Treat-to-Target Strategy over Conventional Treatment with Fixed csDMARD and Corticosteroids: A Multi-Center Randomized Controlled Trial in RA Patients with an Inadequate Response to Conventional Synthetic DMARDs, and New Therapy with Certolizumab Pegol
Source: J Clin Med. 2019 Mar 3;8(3):302. doi: 10.3390/jcm8030302 (PMC6462919; doi:10.3390/jcm8030302)
Supplement: Supplementary file 1 [file jcm-08-00302-s001.pdf]

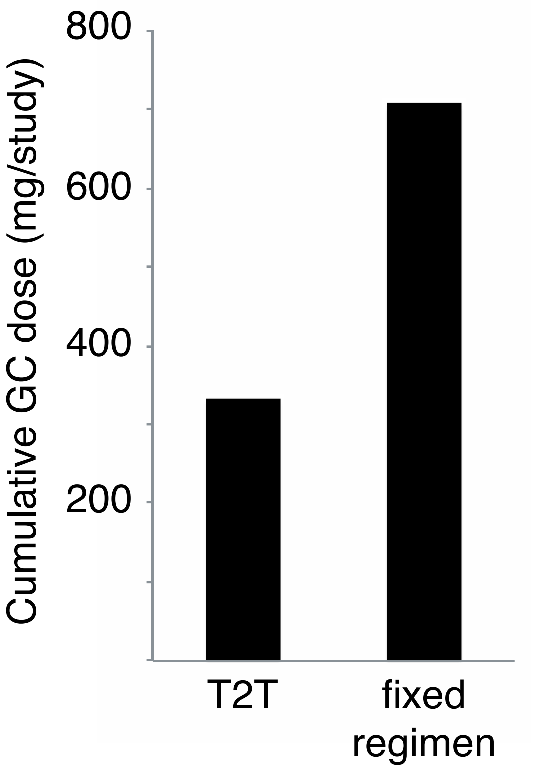


**Figure S1.** Cumulative GC (glucocorticoids) dose during the study period. The average cumulative GC dose was calculated separately for patients treated with T2T or with fixed regimen. The average cumulative GC dose was standardized for prednisolone or equivalent.

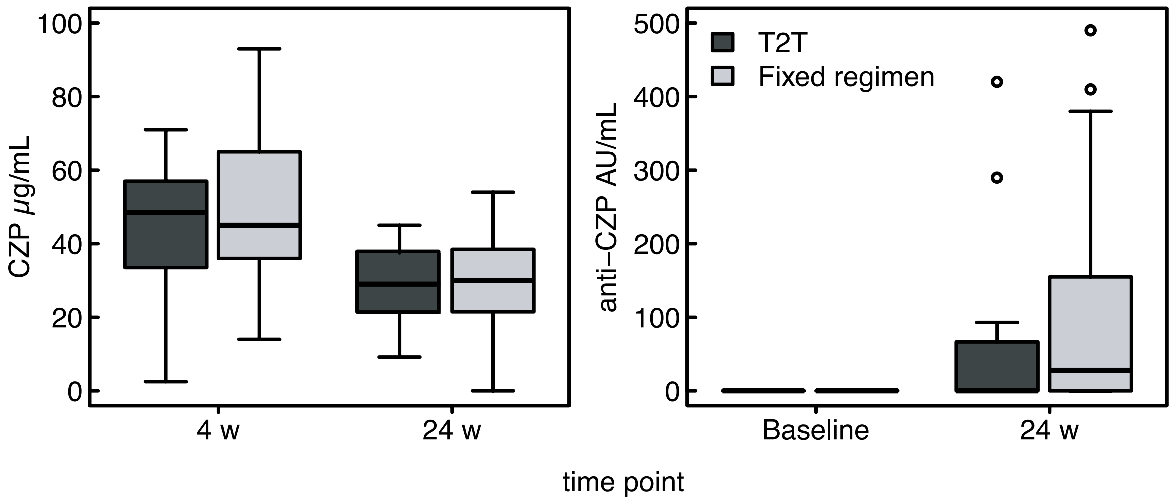


**Figure S2.** (**A**) CZP (certolizumab pegol) levels and were measured at week 4 and week 24. (**B**) CZP anti-drug antibodies were measured at the baseline and week 24. Patients treated with CZP + T2T are shown in dark grey and CZP + fixed regimen in light grey. Data are shown as medians with the 25th and 75th percentile and the standard deviation as error bars. Outliers are implemented as dots; w: week, AU/mL: arbitrary units per milliliter, T2T: treat to target.
